# Variability in prescription drug expenditures explained by adjusted clinical groups (ACG) case-mix: A cross-sectional study of patient electronic records in primary care

**DOI:** 10.1186/1472-6963-8-53

**Published:** 2008-03-04

**Authors:** Alba Aguado, Elisabet Guinó, Bhramar Mukherjee, Antoni Sicras, Josep Serrat, Mateo Acedo, Juan Jose Ferro, Victor Moreno

**Affiliations:** 1 Catalan Institute of Health, SAP Baix Llobregat Centre, Cornella, Barcelona, Spain; 2 Consorci Sanitari Integral, Barcelona, Spain; 3 Catalan Institute of Oncology, IDIBELL. L'Hospitalet, Barcelona, Spain; 4 Department of Biostatistics, School of Public Health, University of Michigan, Ann Arbor, MI, USA; 5 Badalona Serveis Assistencials, Badalona, Spain; 6 Hospital Sant Joan de Deu, Esplugues de Llobregat, Barcelona, Spain; 7 University of Barcelona, School of Medicine Campus Bellvitge, L'Hospitalet, Barcelona, Spain

## Abstract

**Background:**

In view of rapidly increasing prescription costs, case-mix adjustment should be considered for effective control of costs. We have estimated the variability in pharmacy costs explained by ACG in centers using patient electronic records, profiled centers and physicians and analyzed the correlation between cost and quality of prescription.

**Methods:**

We analyzed 65,630 patient records attending five primary care centers in Spain during 2005. Variables explored were age, gender, registered diagnosed episodes of care during 2005, total cost of prescriptions, physician and center. One ACG was assigned to each patient with ACG case-mix software version 7.1. In a two-part model, logistic regression was used to explain the incurrence of drug expenditure at the first stage and a linear mixed model that considered the multilevel structure of data modeled the cost, conditional upon incurring any expense. Risk and efficiency indexes in pharmacy cost adjusted for ACG were obtained for centers and physicians. Spearman rank correlation between physician expenditure, adjusted for ACG, and a prescription quality index was also obtained. Pediatric and adult data were analyzed separately.

**Results:**

No prescription was recorded for 13% of adults and 39.6% of children. The proportion of variance of the incurrence of expenditure explained by ACGs was 0.29 in adults and 0.21 in children. For adults with prescriptions, the variance of cost explained by ACGs was 35.4%, by physician-center was 1.8% and age 10.5% (residual 52.3%). For children, ACGs explained 22.4% of cost and physician-center 10.9% (residual 66.7%). Center efficiency index for adults ranged 0.58 to 1.22 and for children 0.32 to 2.36.

Spearman correlation between expenditure and prescription quality index was -0.36 in family physicians (p = 0.019, N = 41) and -0.52 in pediatricians (p = 0.08, N = 12).

**Conclusion:**

In our setting, ACG is the variable studied that explains more variability in pharmacy cost in adults compared to physician and center. In children there is greater variability among physicians and centers not related to case-mix. In our sites, ACG is useful to profile physicians and centers using electronic records in real practical conditions. Physicians with lower pharmaceutical expenditure have higher scores for a prescription quality index.

## Background

Prescription drug costs are rapidly increasing in most countries. The pharmaceutical expenditure in Catalonia, Spain is 25% of total health cost [1]. Since patient complexity is a major determinant of expenditure, in order to control drug costs more effectively and with equity, methods for case mix adjustment should be considered.

The adjusted clinical groups (ACGs) system was developed at the Johns Hopkins University in Baltimore [2,3]. It estimates individual health status and risk for health service use based on age, gender and diagnoses assigned over a defined time interval, typically one year. Each diagnosis is assigned to one of 32 aggregated diagnostic groups (ADGs). Each ADG is a cluster of similar conditions based on their expected impact on health services resource consumption. Patients are then classified into one ACG based on age, gender and constellation of ADGs.

Even though this system was developed in the USA, it has been used and assessed in other countries. In Spain, most of the studies available have been performed in experimental conditions. There is little information on the utility of the system in real conditions of the daily clinical practice and using the physicians encounter data. Recently, Primary Care centers in the Catalan Institute of Health have substituted patient clinical records from paper to electronic format. This makes it possible to test the ACG system in real conditions.

Physicians are sometimes reluctant to accept a control of their expenditure. It is often argued that a reduction in cost implies a reduced quality of health care. But the selection of low cost health providers as compared with others of higher cost does not necessarily mean health care service is adversely affected [4]. In Spain, older medications are usually cheaper than new drugs and their safety and efficacy profile is often best known. Physicians might accept better a control of their expenditure if they knew that quality is not compromised.

In this study we have three aims: 1) to estimate the variability in prescription drug costs explained by ACG in centers using electronic patient records, 2) to profile centers and physicians after adjusting for ACG, and 3) to analyze the correlation between pharmacy costs adjusted for ACG and quality of prescription.

## Methods

### Setting

This is a cross-sectional observational study. Data were obtained retrospectively from electronic records in five primary care centers. All family physicians and pediatricians working in these centers for the whole year and their patients who attended at least once during the period January to December of 2005 have been studied. The centers are located in Baix Llobregat Centre, close to Barcelona and are referred as center A (assigned population: 21,748), center B (15,848), center C (26,768), center D (14,281) and center E (15,937). These centers have been using exclusively electronic records (software OMI AP version 6.0) for more than 2 years.

### Data retrieval and processing

Data available per patient from electronic records was: encrypted identity number, age, gender, registered diagnosed episodes of care during 2005, drug prescriptions (type and amount), assigned physician (with an encrypted identity code) and center. The diagnosed health problems were coded with the International Classification for Primary Care (ICPC-2). A file with the drug prices from Catsalut, a public agency responsible for contracting and paying for health services in Catalonia, Spain, was used to obtain the cost of prescribed medication per patient (in euros). Electronic printed prescriptions are an overestimation of prescribed drugs that are obtained from the pharmacy store and invoiced to Catsalut. A correction to the patient prescription cost was applied according to the deviation within each center of the prescription cost obtained from electronic files and the real expenditure invoiced.

A conversion (mapping) from ICPC-2 codes to ICD-9-CM has been performed [5]. For this process a working team (a documentalist, 2 clinical doctors and 2 consulters) was created. There were three different groups according to the relations among the two system codes: 1) no relation (one code in ICPC-2 with no equivalent in ICD-9-CM), 2) univocal relation (one code in ICPC-2 had a single correspondence in the ICD-9-CM, this was the optimal situation) and 3) multiple relations (one code in ICPC-2 had several possible codes in ICD-9-CM). For codes in group 1, the documentalist, with the agreement of the rest of the team, codified the descriptors to the closest ICD-9-CM category. For codes in group 3, if all possible ICD codes had the same Adjusted Diagnostic Group, then this ADG was adopted. But if several ADGs were possible, then the most frequent ICD-9-CM code was considered.

From coded diagnoses of episodes, the Johns Hopkins ACG case-mix system software version 7.1 classified subjects into 32 binary (present/absent) Aggregated Diagnosis Groups (ADGs). In a second step, a single ACG category was assigned to each patient based on age, gender and the number and type of ADGs.

Each physician and center had a score for quality of prescription assigned by a computerized program of the Catalan Institute of Health. This institution has developed a quality index for family physicians and for pediatricians according to their prescription. For our study we used the version available for 2005 [see Additional file 1]. In the Catalan Institute of Health official web, the procedures for the construction of the updated 2007 version is available [6]. For family physicians the maximum score is 130 and consists of general and specific indicators that consider mostly the efficacy and safety, and also the efficiency of therapeutic options for the most common conditions in primary care. Global indicators include the use of generics and new drugs, and specific indicators include the use of non steroidal anti-inflammatory drugs, antibiotics and drugs for hypertension, ulcer, hyperlipidemia, asthma, depression, anxiety/insomnia and diabetes. Pediatricians can have a maximum score of 70 according to global indicators that include the use of generics and new drugs, and specific indicators about the use of antibiotics and drugs for asthma.

### Statistical analysis

The distribution of ACG in the population was described separately for pediatric (<15 years) and adults. ACGs with similar mean cost were grouped in resource utilization bands (RUBs) based on quintiles in order to have a reduced number of homogeneous categories. We added one to all values of cost data and then log transformed to reduce the skewness of the distribution and make it close to normal. Only one patient with an extremely high expenditure was excluded from the analysis.

#### Variability in prescription drug expenditures

For univariate analysis, the coefficient of determination (R^2^) derived from linear regression models was calculated for variables expected to explain the variability in drug expenditure. ACG, patient age, physician and center were found to explain a significant proportion of the variance. Physician and center characteristics thought to influence drug expenditure like physician age, gender and assigned population, or center size and teaching activities were explored, but none was significant and were not further considered. Then a multivariate analysis of the sources of variability in pharmacy costs done using variance components analysis derived from linear mixed models that considered the multilevel structure of data (physicians nested within centers). The logarithm of the cost per patient was modeled with the following formula:

log(cost) = α + β_0_·age + β_1_·age^2 ^+ ACG_i _+ physician(center)_j _+ ε_k_

ACG_i _~ N(0, σ^2^_1_)

physician(center)_j _~ N(0, σ^2^_2_)

ε_k _~ N(0, σ^2^_3_)

Age was fitted as a fixed effect, with linear and quadratic terms. ACG and physician (nested within center) were considered random effects. The values observed were supposed to follow a normal distribution with zero mean and a given variance. These variances (σ^2^_1 _and σ^2^_2_) were the parameters of interest for the ACG and physician variables. The residual variance was also a relevant term, because indicated the amount of variability unexplained by these terms. The variances per se were not important, because they depend on the actual data observed. We calculated the relative contribution of each term to the total variance as indicator of the importance of the variable in the variability of pharmaceutical expenditure.

Since the calculation of ACGs considered age and gender, including all these terms in the model would make difficult the interpretation due to colinearity. Gender was not relevant in explaining cost, and we decided to exclude this term, which would have a zero parameter. For age, we first fitted a linear model with ACG as random effect and calculated the residuals for each subject. The residuals can be interpreted as the variability in age not accounted for already in the ACGs terms. Then we used these residuals in a linear and quadratic term as fixed effects in the models to explore the variability in cost. This way, the variance component for age should be interpreted as that not already explained by ACG. These models were estimated using the restricted maximum likelihood method.

A two-part model was also used. In a first step, a binary logistic regression using ACG as a covariate was applied to predict the incurrence of drug expenditure. Nagelkerke R^2 ^was used to estimate the proportion of variance explained. We chose this measure knowing its limitations as an approximation to the coefficient of determination in a linear model. In logistic regression models the concept of residual variance is not easy to define because the response variable is binary. These models are fitted by maximum likelihood and the likelihood value can be used as a relative measure of model fit. The problem is that, when modeling individual data, as in our study, the actual likelihood value depends on sample size and has no interpretation. Nagelkerke [7] defined an equivalent value to the coefficient of determination based on the ratio of likelihoods between the "null" model (with only a constant) and fitted model, appropriately corrected to have a maximum of 1. This coefficient can be interpreted as the goodness of the model in predicting the response from the covariates, similar to R^2 ^in linear regression. We have assumed approximate additivity in the proportion of variance explained by multiple factors, which is appropriate when these are not too large.

In a second step of the two part model, the variability in pharmacy costs restricted to patients with prescriptions were estimated using variance components analysis of linear mixed models as before. ACGs with less than 30 subjects were excluded from the analysis.

#### Profiling of centers and physicians

Risk and efficiency indexes in pharmacy costs, adjusted for ACG, were obtained for centers and physicians. Risk index or morbidity burden was calculated for each center and physician. It shows the complexity of visited population in relation to a standard and is calculated as the ratio between the predicted mean pharmaceutical cost considering the ACG distribution and the mean pharmaceutical cost of the whole population studied. Efficiency index compares pharmaceutical expenditure among centers and physicians assuming similar population complexity. It is calculated as the ratio between the observed mean expenditure and the predicted mean expenditure adjusted for ACG. Expenditure of centers and physicians were compared after adjusting for ACG using linear mixed models.

The Spearman rank correlation between physician pharmacy cost adjusted for ACG and the prescription quality index was calculated.

## Results

### Description of population and variables

**Figure 1:**
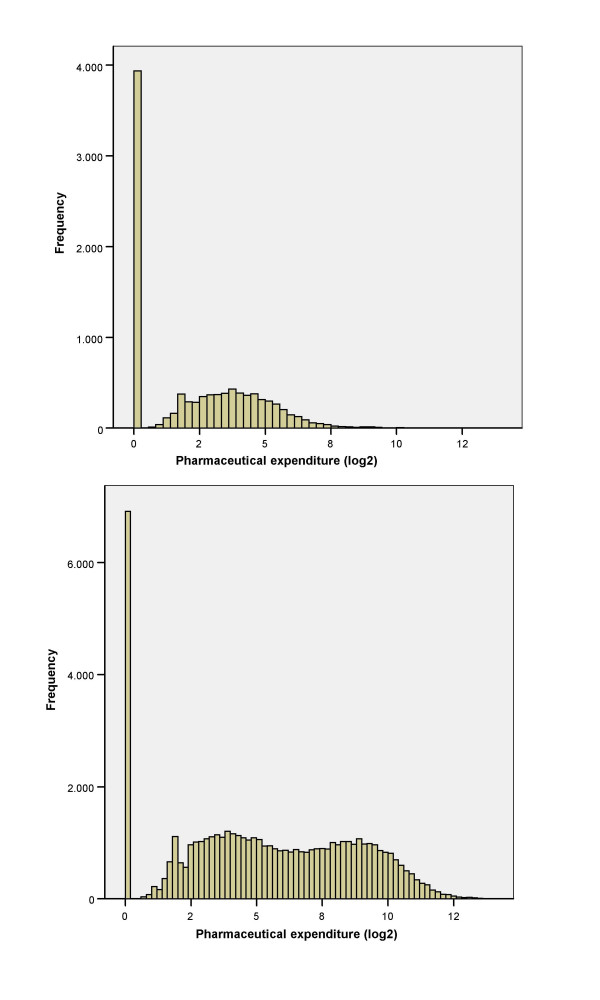
**Distribution of pharmaceutical expenditure**. Distribution of prescription drug expenditure in pediatric population (above) and adults (below).

A total of 55,971 adult and 9,659 pediatric patients have been studied (table 1). The distribution of logarithmic prescription drug expenditure is shown in figure 1. In 13.1% of adult patients and 39.6% of children there was no expenditure. The expenditure of drug prescriptions obtained from the pharmacy store and invoiced to the health system was in average 24.7% lower than the expenditure calculated from the printed prescriptions according to the patient electronic records.

**Table 1:** Characteristics of the population.

Center	Patients		Median age	Pharmaceutical expenditure Median (euros)		Prescription quality score	
			
	Adults	Pediatrics		Adults	Pediatrics	Adults	Pediatrics
A	13043	1940	42	34.5	7.2	106	62
B	9648	1644	43	42.5	2.8	86	29
C	16791	3045	38	36.1	14.6	77	11
D	8413	1671	40	36.5	0	90	62
E	8076	1359	43	44.8	0	63	66

Total	55971	9659	40	37.8	3.79		

A total of 254,830 ADGs were assigned (52,909 in center A, 64,639 in center B, 67,213 in center C, 35,987 in center D and 34,082 in center E). After ranking ACGs by frequency, 75% of patients were distributed in the first 19 (partially listed in table 2) and 90% in the first 34. ACGs have been grouped in 6 homogeneous resource utilization bands (RUBs) (table 3).

**Table 2:** More frequent ACGs and ACGs with more expenditure

ACG	Description	N	%	cumulate%
300	Acute Minor, Age 6+	7087	10.8	10.8
4100	2–3 Other ADG Combinations, Age 35+	6544	10	20.8
4910	6–9 Other ADG Combinations, Age 35+, 0–1 Major ADGs	4948	7.5	28.3
2100	Acute Minor/Likely to Recur, Age 6+, w/o Allergy	3763	5.7	34
4410	4–5 Other ADG Combinations, Age 45+, no Major ADGs	2878	4.4	38.4
1800	Acute Minor/Acute Major	2799	4.3	42.7
4420	4–5 Other ADG Combinations, Age 45+, 1 Major ADGs	2444	3.7	46.4
500	Likely to Recur, w/o Allergies	2333	3.6	50
3600	Acute Minor/Acute Major/Likely to Recur/Chronic Medical: Stable	2102	3.2	53.2
400	Acute Major	1860	2.8	56
3200	Acute Minor/Acute Major/Likely to Recur, Age 12+, w/o Allergy	1859	2.8	58.8
2300	Acute Minor/Chronic Medical: Stable	1704	2.6	61.4
1600	Preventive/Administrative	1522	2.3	63.7
4310	4–5 Other ADG Combinations, Age 18 to 44, no Major ADGs	1380	2.1	65.8
4920	6–9 Other ADG Combinations, Age 35+, 2 Major ADGs	1342	2	67.8
2500	Acute Minor/Psychosocial, w/o Psychosocial Unstable	1282	2	69.8
3500	Acute Minor/Likely to Recur/Psychosocial	1188	1.8	71.6

ACG	Description		mean expenditure	RW

4940	6–9 Other ADG Combinations, Age 35+, 4+ Major ADGs		1674.5	6.1
5060	10+ Other ADG Combinations, Age 18+, 3 Major ADGs		1519.5	5.5
5050	10+ Other ADG Combinations, Age 18+, 2 Major ADGs		1397.4	5.1
4930	6–9 Other ADG Combinations, Age 35+, 3 Major ADGs		1358.6	4.9
5070	10+ Other ADG Combinations, Age 18+, 4+ Major ADGs		1278.6	4.6
4920	6–9 Other ADG Combinations, Age 35+, 2 Major ADGs		1049.3	3.8
5040	10+ Other ADG Combinations, Age 18+, 0–1 Major ADGs		924.0	3.3
4430	4–5 Other ADG Combinations, Age 45+, 2+ Major ADGs		884.8	3.2
1400	Psychosocial, w/Psychosocial Unstable, w/o Psychosocial Stable		755.1	2.7
4420	4–5 Other ADG Combinations, Age 45+, 1 Major ADGs		741.2	2.7
4910	6–9 Other ADG Combinations, Age 35+, 0–1 Major ADGs		703.0	2.5
800	Chronic Medical: Unstable		630.6	2.3
2700	Acute Minor/Psychosocial, w/Psychosocial Unstable/Psychosocial Stable		617.7	2.2
4730	6–9 Other ADG Combinations, Males, Age 18 to 34, 2+ Major ADGs		615.1	2.2

More frequent ACG categories (above) and ACG with higher relative weights (RW) for pharmaceutical expenditure (below).

**Table 3:** Distribution in Resource Utilization Bands (RUBs)

	**RUB 1**	**RUB 2**	**RUB 3**	**RUB 4**	**RUB 5**	**RUB 6**
Median expenditure	0	1.9	7.4	27.8	135	468.3

Center A	5.6	5.2	38.6	15.6	23.4	11.6
Center B	3.6	1.3	25.3	22.9	17.0	30.0
Center C	2.1	4.3	41.1	20.3	17.2	14.9
Center D	4.4	5.0	42.4	16.5	20.1	11.8
Center E	0.4	2.7	35.2	21.9	21.1	18.7

Total	3.2	3.8	37.2	19.3	19.6	16.8

Percent distribution of patients in resource utilization bands. Median pharmaceutical expenditure (in euros) for each band.

### Explained variability in pharmaceutical expenditure

When considering individual variables (univariate analysis), the proportion of variance explained (R^2^) of pharmaceutical expenditure was, for adult patients: 0.31 for age, 0.01 for gender, 0.03 for physician and 0.39 for ACG. For pediatric patients R^2 ^values were: 0.04 for age, 0 for gender, 0.33 for physician and 0.19 for ACG. Between centers, R^2 ^for ACGs in adult population showed little variability (0.32 to 0.45) while in pediatric patients the proportion of cost explained by ACGs was smaller and more disperse between centers (0.09 to 0.39).

In table 4 the results of applying linear mixed models to estimate variance components for the whole population for adults and children are presented. It also presents the results from the two-part model, in which first the incurrence of expenditure was modeled with logistic regression followed by a linear mixed model to estimate the components of variance of the level of cost conditional upon incurring any expense. It is noteworthly that while heterogeneity among physicians explains little of adult expenditure, this factor is very relevant for pediatric expenditure, and the proportion of explained variance is larger for the incurrence of expenditure than for the cost. Supplementary tables give detailed estimates for these models overall and for each center [see Additional file 2].

**Table 4:** Cost modeling approaches for prediction of pharmaceutical expenditure.

	**Total population**			**Two-part model**			
				
				**Expenditure incurrence**	**Level of expenditure**		
	
	**Linear mixed models**			**Logistic regression**	**Linear mixed models**		
**Adult population**							
**Fixed effects**	**V**	**SE**	**%V**	**%EV**	**V**	**SE**	**%EV**
Age*			1.4%	0.6%	10.5%		10.5%
**Random factors**							
ACG	5.01	0.89	44.7%	28.8%	2.78	0.49	35.4%
Physician (center)	0.27	0.06	2.4%	1.5%	0.14	0.03	1.8%
Residual	5.77	0.04	51.5%	69.1%	4.11	0.03	52.3%
**Total**	11.21		100%	100%	7.85		100%

**Pediatric population**							
**Fixed effects**	**V**	**SE**	**%V**	**%EV**	**V**	**SE**	**%EV**
Age*			0.1%	0.4%			0.0%
**Random factors**							
ACG	1.22	0.28	22.2%	20.6%	0.66	0.17	22.4%
Physician (center)	1.62	0.69	29.5%	38.8%	0.32	0.14	10.9%
Residual	2.66	0.04	48.3%	40.2%	1.98	0.04	66.7%
**Total**	5.51		100%	100%	2.96		100%

Statistical tests applied to the total population and two-part model. Linear mixed models include: estimation of variance (V), standard error (SE) and percentage of explained variance (%EV).

* Age fixed effects include linear and quadratic terms.

**Figure 2:**
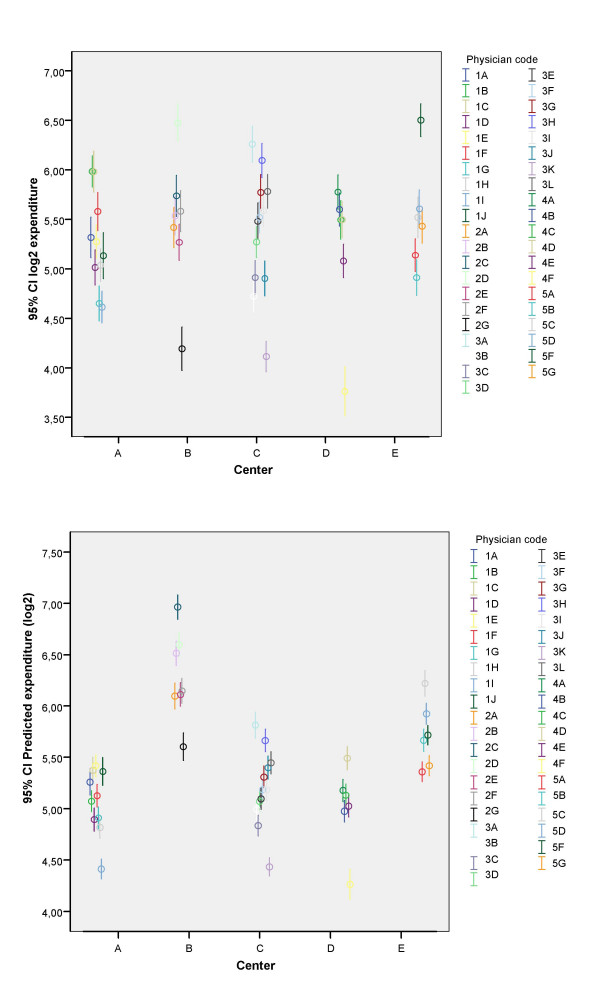
**Family physicians pharmaceutical expenditure**. Prescription drug expenditure for family physicians, crude (above) and adjusted for ACGs (below).

The observed and expected expenditure after adjusting for ACG for each of the centers and the risk and efficiency indexes are presented in table 5. In figure 2 the crude and adjusted expenditures for each physician are represented. It is apparent that the adjustment for ACGs reduces the variability in mean expenditure.

**Table 5:** Risk and efficiency index for centers.

**Family physicians**				
Center	Observed expenditure	Expected expenditure	Risk Index	Efficiency Index

A	37.94	32.80	0.78	1.16
B	46.14	79.84	1.90	0.58
C	41.18	36.62	0.87	1.12
D	41.80	34.21	0.81	1.22
E	46.85	51.57	1.23	0.91

Total	41.99	41.99	1.00	1.00

**Pediatricians**				

Center	Observed expenditure	Expected expenditure	Risk Index	Efficiency Index

A	7.28	5.00	0.97	1.46
B	4.34	6.02	1.16	0.72
C	13.09	5.56	1.07	2.36
D	1.25	3.93	0.76	0.32
E	2.70	5.45	1.05	0.50

Total	5.18	5.18	1.00	1.00

Mean pharmaceutical expenditure per visited patient, observed and expected according to ACGs distribution (in euros).

### Correlation between physician pharmaceutical expenditure and quality score

When using crude expenditure, in family physicians the Spearman rank correlation was -0.18 (N = 41, p = 0.26) and in pediatricians it was -0.54 (N = 12, p = 0.07). After adjusting for ACGs using a linear model, the correlation in adults was -0.36 (p = 0.019) and in pediatricians -0.52 (p = 0.08).

## Discussion

In this study we show that patient case-mix measured with ACGs is the major determinant of pharmaceutical expenditure variability in our setting. Adjustment for ACGs allows a much fair comparison of expenditures among centers and physicians. We also show that pharmaceutical expenditure correlates negatively with a prescription quality index more strongly when adjusted for ACGs, which can be interpreted as evidence that, when complexity of the patients is accounted for, better quality of prescription is associated with lower expenditure.

In this analysis adult and pediatric data have been analyzed separately because their distribution is different and the proportion of expenditure variance explained for the main factors is also different. In adults, the expenditure is higher. A significant percentage of patients attended the centers but had no prescription (13%, figure 1). The distribution of the logarithmic expenditure looks like a mixture of two overlapped normal populations. Possibly this shape is due to the patients with chronic medication that have higher costs, but we have no data to prove it because this variable was not available. In pediatric patients, the proportion attending the centers without prescription is higher, 40% and the distribution of the logarithmic expenditure looks like a single normal population.

Healthcare cost data usually have mixed distributions with a high percentage of non-users and a right-skewed distribution for users [8]. Two-part modeling has been used to address the issue of a mixed distribution of data. This approach first estimates the probability of incurring any cost and subsequently models the amount of cost conditional on having incurred any cost [9]. ACG explain 28.8% of the variability in the incurrence of drug prescriptions in adults and 20.6% in children. In subjects with prescriptions, the variability in expenditure explained by ACG accounts for 35.4% in adults and 22.4% in children. We should emphasize that the results obtained here are site specific and need not generalize to other sites with different patient population profiles, non-primary care settings or structure of care provision. However, these results are similar to those obtained by other authors in real practice conditions. Orueta [10] in the Basque Country, Spain proved ACG was useful in real conditions of daily practice to explain 50% of the variance in visits to primary care physicians, 25 – 40% of prescriptions, 25 – 30% of referrals and requests of laboratory tests and 14–16% of radiographs. The coefficients of determination remained almost invariable after the addition of hospital diagnosis or correction of coding errors by the research team. Sicras [11] found that 20% of the variability in pharmacy costs was explained by ACGs in a non-public primary care health center in Catalonia, Spain, using a retrospective setting.

Only one patient with extremely high expenditure was excluded from the analysis. No other data were truncated to remove the effect of outliers. This would have improved the fit of the models. But a small proportion of the population accounts for a large amount of pharmaceutical expenditure [12]. So if data are not truncated, the ability to predict pharmacy costs for the patients with the highest expenditures that often contribute a disproportionate amount of total cost is preserved.

More than half of the variability in prescription drug expenditure still remains to be explained. Context variables, summarized in center, show little impact in variability. And physician related factors are only relevant in pediatric population. Some other variables not recorded and measurement error may play a role in the observed variability. The accumulation of different kind of errors may be heterogeneous among centers and professionals. The main sources of errors are: deficient quality in patient record files, errors in diagnostic coding and in recoding from ICPC-2 to ICD-9-MC. Our source of information for prescription cost was the patient electronic files, which records information of printed prescriptions. This is an overestimation of real cost invoiced to the health system for several reasons: a prescription might be printed several times because of technical problems. Also, not all patients with a prescription go to the pharmacy store to get the drug. As a result of this, the real pharmacy cost invoiced to the health system is not as high as that obtained from the patient electronic file. In our study we estimated a deviation of 25%, and an appropriate correction was applied when estimating the sources of variability. Although the deviation is important, and it is a limitation of the study, we cannot analyse in detail these unfilled prescriptions because the real cost per person invoiced to the health system is not available. Only the global deviation per center was available and that is what we used for correction.

ACG system has proved useful in the profiling of centers and physicians when data are obtained from electronic records in real conditions of usual practice. It is possible to identify physicians and centers with high expenditure but with a good efficiency index after adjusting for case-mix. Also some centers and professionals have an apparent low cost but considering their case mix, a lower expenditure would be expected.

In center B, the median expenditure per visited patient in adults is one of the highest. After adjusting for ACG, it becomes apparent the population visited has more burden of morbidity (risk index 1.9) and has the best efficiency index (0.58). On the other hand, center A has the lowest median expenditure per patient in adults. After adjusting for ACG, the population has the lowest burden of morbidity (risk index: 0.78) and the efficiency is less than satisfactory (efficiency index: 1.16). When considering the median expenditure per pediatric patient, center C has an extremely high value. After adjusting for ACG, the burden of morbidity of their patients is slightly above average and the efficiency index is very poor (an index of 2.36). Similar observations can be performed considering each physician's prescription. Other authors have also used ACG system for profiling of physicians and showed similar results [13,14].

Sites D and E have lower paediatric expenditure with a median of 0 per paediatric patient (table 1). As it is evident in table 5, the mean observed cost for paediatric patient in sites D and E patient is lower than the expected expenditure according to ACG distribution, and so the efficiency index in these centers is better. Globally in our setting, 40% of total children had no prescription and there was a high variability in cost explained by physician and center. In paediatric care, the case-mix does not account for so much variability in prescription cost as in adults. Most of the children visited in primary care setting have not serious diseases and there seems to be a different prescription approach according to the physician involved.

It is important to check that the quality of care is not compromised when expenditure is controlled. In fact, family physicians with higher quality scores have lower expenditure and this correlation is improved after adjusting for ACGs. The number of pediatricians studied is very low (N = 12), but still the Spearman rank correlation approaches a significant value. In this case, adjusting for ACG does not improve the correlation. Medications with a long time in the market usually have a lower cost than new drugs. Older medications often have a better known safety and efficacy profile. Because quality is not reduced when prescription cost is low, it might be easier for physicians to accept a control of their expenditure.

## Conclusion

In our setting, case-mix, measured with the ACG system, is the variable studied that explains more variability in prescription drug expenditure in adults. In pediatric population there is greater variability among physicians and centers which is not related to case-mix. The implementation of clinical guidelines might be helpful to reduce this variability.

For our sites, ACG is a useful tool to analyze efficiency and compare physicians and centers when data are obtained from electronic records in real conditions of usual practice. It is possible to identify physicians and centers with high expenditure but with a good efficiency index after adjusting for case-mix, and also centers and professionals with apparent low cost but considering their case mix, a lower expenditure would be expected. Physicians with lower pharmaceutical expenditure have higher scores for the prescription quality index studied and in adult population the correlation increases when expenditure is adjusted for ACG.

## Competing interests

The author(s) declare that they have no competing interests.

## Authors' contributions

AA designed the study, applied the ACG software, carried out the first calculations of the results and drafted the manuscript. VM rechecked and revised statistical analysis with assistance from BM. EG processed and prepared the databases before being analyzed. AS and JS converted the ICPC-2 codes into ICD-9-MC codes. MA extracted and organized the data from the electronic records. JF collected the prescription cost invoiced to the health system and the physicians' prescription quality scores. All authors read and approved the final manuscript.

## Pre-publication history

The pre-publication history for this paper can be accessed here:



## Supplementary Material

Additional file 1 Appendix 1. Criteria for the prescription quality index. Click here for file

Additional file 2 Supplementary tables. Detailed tables with variance components model parameters for adult population and paediatric population, spitted by centre. Click here for file
