# Supplementary material for: Variability in prescription drug expenditures explained by adjusted clinical groups (ACG) case-mix: A cross-sectional study of patient electronic records in primary care
Source: BMC Health Serv Res. 2008 Mar 4;8:53. doi: 10.1186/1472-6963-8-53 (PMC2292169; doi:10.1186/1472-6963-8-53)
Supplement: Additional file 1 — Appendix 1. Criteria for the prescription quality index. [file 1472-6963-8-53-S1.doc]

**Appendix 1.**

PRESCRIPTION QUALITY INDEX FOR FAMILY PHYSICIANS.

(Maximal score: 130)

Global indicators: goal scores

Percentage of use of drugs of proven efficacy >87% 3

>90% 5

Percentage of use of generics >9% 5

>12% 10

>15% 15

Percentage of prescription of new drugs without therapeutic advantage according to the Committee of evaluation of New Drugs <1.8% 5

<1.6% 10

<1.4% 15

Specific indicators:

1. Antihypertensive drugs (HT):

Percentage of diuretic and beta blockers from total HT: >40% 4

>35% 2

Percentage of alpha1 blockers from total HT: <2% 1

Percentage of angiotensin 2 antagonist from total of ACE inhibitors and angiotensin 2 antagonist: < 20% 4

< 25% 2

Percentage of recommended HT from total HT: > 72% 6

> 67% 3

(Recommended HT: atenolol, bisoprolol, metoprolol, hydroclorthiazide, amiloride, clortalidone, captopril, enalapril, lisinopril, diltiazem, verapamil, losartan, amlodipine).

2. Drugs for gastroduodenal ulcus (ULC):

Definite daily doses of ULC/1000 inhabitants standardized for older than 65/day:

< 17 6

< 20 3

Percentage of recommended ULC from total ULC: > 90% 4

> 85% 2

(Recommended ULC: almagate, algedrate, magaldrate and other aluminium and magnesium salts, ranitidine, omeprazole, misoprostol).

3. Non-steroidal anti-inflammatory drugs (NSAID)

Definite daily doses of NSAID/1000 inhabitants standardized for older than 65/day:

< 11 4

< 13 2

Percentage of piroxicam and tenoxicam from total NSAID:

< 4% 2

Percentage of recommended NSAID from total NSAID: > 75% 4

> 70% 2

(Recommended NSAID: ibuprofen, naproxen, diclofenac).

4. Antibiotics (AB):

Definite daily doses of AB/1000 inhabitants standardized for older than 65/day:

< 3.5 6

< 5 4

Percentage of penicillin from total AB: > 60% 2

> 55% 1

Percentage of recommended AB from total AB: > 75% 2

> 70% 1

(Recommended AB: amoxicillin, amoxicillin-clavulanic, penicillin G, penicillin V, cloxacillin, eritromicin, claritromicin, pipemidic, norfloxacin, fosfomicin-tromatamol).

5. Drugs for hyperlipidemia (LIP):

Percentage of recommended LIP from total LIP: > 70% 10

> 65% 5

(Recommended LIP: colestiramine, colestipol, gemfibrozile, sinvastatine, lovastatine)

6. Drugs for asthma (ASM):

Percentage of recommended ASM from total ASM: > 76% 10

> 71% 5

(Recommended ASM: salbutamole, terbutaline, salmeterole, formoterole, ipratropium bromure, budesonide, beclometasone)

7. Drugs for depression (DEP):

Percentage of recommended DEP from total DEP: > 70% 10

> 65% 5

(Recommended DEP: nortriptiline, imipramine, clomipramine, fluoxetine, paroxetine, citalopram)

8. Drugs for anxiety / insomnia (ANX):

Definite daily doses of ANX/1000 inhabitants standardized for older than 65/day:

< 20 5

< 22 3

Percentage of recommended ANX from total ANX: > 87% 5

> 82% 3

(Recommended ANX: alprazolam, lormetazepam, diazepam, clorazepate, lorazepam).

9. Oral drugs for diabetes (DB):

Percentage of recommended DB from total DB: > 80% 5

> 75% 3

(Recommended DB: glibenclamide, gliclazide, metformine).

PRESCRIPTION QUALITY INDEX FOR PEDIATRICIANS. (Maximal score: 70)

Global indicators: goal scores

Percentage of use of drugs of proven efficacy >90% 5

>87% 3

Percentage of use of generics >9% 5

>12% 10

>15% 15

Percentage of prescription of new without therapeutic advantage according to the Committee of evaluation of New Drugs <0.7% 7

<0.9% 4

<1.1% 2

Specific indicators:

1. Antibiotics (AB):

Definite daily doses of AB/1000 inhabitants/day: < 8 8

< 9 6

Percentage of penicillin from total AB: > 75% 6

> 70% 4

Percentage of macrolides from total AB: < 12% 5

< 15% 3

Percentage of recommended AB from total AB: > 87% 6

> 82% 4

(Recommended AB: amoxicillin, amoxicillin-clavulanic, penicillin G, penicillin V, cloxacillin, cefuroxime-axetil, eritromicine, claritromicine, josamine, cefadroxile).

2. Drugs for asthma (ASM):

Percentage of inhalated ASM from total ASM: > 90% 6

> 85% 3

Percentage of recommended ASM from total ASM: > 80% 9

> 75% 5

(Recommended ASM: salbutamole, terbutaline, salmeterole, formoterole, budesonide).

The goals for each indicator have been fixed considering the physicians with better scores.

The units for all indicators are definite daily dosages, except for global indicators which are expressed in packages.
