# Supplementary material for: Variability in prescription drug expenditures explained by adjusted clinical groups (ACG) case-mix: A cross-sectional study of patient electronic records in primary care
Source: BMC Health Serv Res. 2008 Mar 4;8:53. doi: 10.1186/1472-6963-8-53 (PMC2292169; doi:10.1186/1472-6963-8-53)
Supplement: Additional file 2 — Supplementary tables. Detailed tables with variance components model parameters for adult population and paediatric population, spitted by centre. [file 1472-6963-8-53-S2.pdf]

Supplementary table 1
Multivariate components of variance analysis
Adult population

Total population

	Parameter	% variance	Estimate	Std. Error	Wald	Sig.	95% CI lower	95% CI upper
Center A								
Fixed	Intercept		4.95	0.28	17.37	0.00	4.38	5.52
	age		0.66	0.01	77.00	0.00	0.64	0.68
	age squared		-0.02	0.00	-6.67	0.00	-0.03	-0.02
Variance		1.4%	0.15					
Random	Residual	51.5%	5.77	0.04	162.06	0.00	5.70	5.84
	ACG	44.7%	5.01	0.89	5.64	0.00	3.54	7.10
	MED	2.4%	0.27	0.06	4.39	0.00	0.17	0.43
Total variance		100.0%	11.21					

Two part model
Expenditure incurrence

		% Variance	B	S.E.	Wald	Sig.	Exp(B)
Center A							
Fixed	age		0.13	0.01	175.53	0.00	1.14
	age squared		-0.02	0.00	24.84	0.00	0.98
	Intercept		1.91	0.02	15769.60	0.00	6.75
Variance		0.6%					
Random	MED	1.5%					
	ACG	28.8%					
	Residual	69.1%					
	Total	100.0%					

Level of expenditure

	Parameter	% variance	Estimate	Std. Error	Wald	Sig.	95% CI lower	95% CI upper
Center A								
Fixed	Intercept		5.76	0.21	26.85	0.00	5.34	6.19
	age		0.62	0.01	79.29	0.00	0.60	0.63
	age squared		-0.02	0.00	-6.29	0.00	-0.03	-0.01
Variance		10.5%	0.82					
Random	Residual	52.3%	4.11	0.03	151.04	0.00	4.06	4.16
	ACG	35.4%	2.78	0.49	5.65	0.00	1.96	3.93
	MED	1.8%	0.14	0.03	4.34	0.00	0.09	0.23
Total variance		100.0%	7.85					

Supplementary table 2
Multivariate components of variance analysis
Pediatrics

Total population

	Parameter	% variance	Estimate	Std. Error	Wald	Sig.	95% CI lower	95% CI upper
Center A								
Fixed	Intercept		2.35	0.40	5.84	0.00	1.49	3.20
	age		-0.29	0.05	-5.74	0.00	-0.39	-0.19
	age squared		0.04	0.04	0.93	0.35	-0.04	0.11
Variance		0.1%	0.00					
Random	Residual	48.3%	2.66	0.04	70.29	0.00	2.58	2.73
	ACG	22.2%	1.22	0.28	4.43	0.00	0.79	1.90
	MED	29.5%	1.62	0.69	2.34	0.02	0.70	3.75
Total variance		100.0%	5.51					

Two part model
Expenditure incurrence

		% Variance	B	S.E.	Wald	Sig.	Exp(B)
Center A							
Fixed	age		-0.27	0.06	18.66	0.00	0.76
	age squared		-0.30	0.13	5.50	0.02	0.74
	Intercept		0.46	0.02	338.34	0.00	1.58
Variance		0.4%					
Random	MED	38.8%					
	ACG	20.6%					
	Residual	40.2%					
	Total	100.0%					

Level of expenditure

	Parameter	% variance	Estimate	Std. Error	Wald	Sig.	95% CI lower	95% CI upper
Center A								
Fixed	Intercept		3.66	0.21	17.62	0.00	3.23	4.08
	age		-0.09	0.06	-1.58	0.11	-0.20	0.02
	age squared		-0.08	0.06	-1.27	0.20	-0.20	0.04
Variance		0.0%	0.00					
Random	Residual	66.7%	1.98	0.04	54.52	0.00	1.91	2.05
	ACG	22.4%	0.66	0.17	3.95	0.00	0.40	1.09
	MED	10.9%	0.32	0.14	2.30	0.02	0.14	0.76
Total variance		100.0%	2.96					

Supplementary table 3
Parameter estimates by center for Total population
Adults

	Parameter	% variance	Estimate	Std. Error	Wald	Sig.	95% CI lower	95% CI upper
Center A								
Fixed	Intercept		5.07	0.31	16.53	0.00	4.46	5.68
	age		0.64	0.02	34.68	0.00	0.60	0.67
	age squared		-0.01	0.01	-1.61	0.11	-0.02	0.00
Variance		5.0%	0.55					
Random	Residual	50.7%	5.58	0.07	79.26	0.00	5.45	5.72
	ACG	43.1%	4.75	0.93	5.10	0.00	3.23	6.97
	MED	1.2%	0.13	0.06	2.05	0.04	0.05	0.34
Total variance		100.0%	11.01					
Center B								
Fixed	Intercept		4.55	0.33	13.69	0.00	3.89	5.22
	age		0.76	0.02	35.75	0.00	0.72	0.80
	age squared		-0.06	0.01	-6.60	0.00	-0.08	-0.04
Variance		6.5%	0.81					
Random	Residual	51.7%	6.44	0.10	63.46	0.00	6.24	6.64
	ACG	40.7%	5.07	1.01	5.03	0.00	3.44	7.49
	MED	1.1%	0.14	0.09	1.66	0.10	0.04	0.46
Total variance		100.0%	12.47					
Center C								
Fixed	Intercept		5.45	0.28	19.32	0.00	4.88	6.01
	age		0.58	0.02	38.85	0.00	0.55	0.61
	age squared		-0.01	0.01	-1.82	0.07	-0.02	0.00
Variance		7.5%	0.81					
Random	Residual	48.7%	5.23	0.06	91.10	0.00	5.12	5.34
	ACG	43.1%	4.62	0.84	5.48	0.00	3.23	6.61
	MED	0.7%	0.08	0.03	2.23	0.03	0.03	0.19
Total variance		100.0%	10.74					
Center D								
Fixed	Intercept		5.24	0.31	17.03	0.00	4.62	5.85
	age		0.64	0.02	27.29	0.00	0.60	0.69
	age squared		-0.01	0.01	-1.25	0.21	-0.03	0.01
Variance		4.5%	0.49					
Random	Residual	48.9%	5.36	0.09	59.55	0.00	5.19	5.54
	ACG	46.3%	5.08	1.03	4.91	0.00	3.41	7.57
	MED	0.3%	0.04	0.03	1.35	0.18	0.01	0.16
Total variance		100.0%	10.97					
Center E								
Fixed	Intercept		4.96	0.35	14.02	0.00	4.23	5.69
	age		0.68	0.02	30.65	0.00	0.64	0.72
	age squared		-0.03	0.01	-3.01	0.00	-0.04	-0.01
Variance		1.1%	0.13					
Random	Residual	57.9%	6.57	0.10	63.08	0.00	6.37	6.78
	ACG	38.3%	4.35	0.86	5.06	0.00	2.95	6.41
	MED	2.7%	0.30	0.19	1.55	0.12	0.09	1.07
Total variance		100.0%	11.35					

Supplementary table 4
Parameter estimates by center for Total population
Pediatrics

							95% CI	
	Parameter	% variance	Estimate	Std. Error	Wald	Sig.	lower	upper
Center A								
Fixed	Intercept		2.78	0.25	11.25	0.00	2.28	3.29
	age		-0.50	0.12	-4.33	0.00	-0.73	-0.28
	age squared		0.10	0.05	2.31	0.02	0.02	0.19
Variance		0.6%	0.03					
Random	Residual	53.0%	2.37	0.08	31.21	0.00	2.23	2.53
	ACG	45.8%	2.05	0.49	4.16	0.00	1.28	3.29
	MED	0.6%	0.03	0.03	0.88	0.38	0.00	0.25
Total variance		100.0%	4.48					
Center B								
Fixed	Intercept		2.10	0.99	2.13	0.27	-9.15	13.34
	age		-0.17	0.12	-1.41	0.16	-0.41	0.07
	age squared		0.09	0.19	0.46	0.64	-0.29	0.46
Variance		0.03%	0.00					
Random	Residual	53.0%	3.02	0.10	29.12	0.00	2.83	3.23
	ACG	13.8%	0.79	0.21	3.75	0.00	0.47	1.33
	MED	33.2%	1.89	2.68	0.71	0.48	0.12	30.44
Total variance		100.0%	5.71					
Center C								
Fixed	Intercept		3.73	0.26	14.25	0.00	3.20	4.26
	age		-0.27	0.10	-2.55	0.01	-0.47	-0.06
	age squared		-0.04	0.30	-0.15	0.88	-0.63	0.54
Variance		0.5%	0.02					
Random	Residual	53.2%	2.82	0.07	39.12	0.00	2.68	2.96
	ACG	46.2%	2.45	0.60	4.11	0.00	1.52	3.94
	MED	0.1%	0.01	0.01	0.65	0.52	0.00	0.16
Total variance		100.0%	5.30					
Center D								
Fixed	Intercept		0.45	0.08	5.82	0.00	0.28	0.62
	age		0.06	0.08	0.75	0.45	-0.09	0.20
	age squared		-0.02	0.18	-0.10	0.92	-0.36	0.33
	Age	0.1%	0.00					
Random	Residual	89.9%	1.00	0.03	28.83	0.00	0.94	1.07
	ACG	9.9%	0.11	0.05	2.08	0.04	0.04	0.28
	MED	0.2%	0.00	0.00	0.42	0.68	0.00	0.20
Total variance		100.0%	1.12					
Center E								
Fixed	Intercept		1.53	0.87	1.77	0.32	-8.44	11.51
	age		-0.24	0.13	-1.78	0.08	-0.50	0.02
	age squared		-0.25	0.33	-0.74	0.46	-0.90	0.41
	Age	0.3%	0.01					
Random	Residual	59.2%	2.81	0.11	25.89	0.00	2.60	3.03
	ACG	9.6%	0.46	0.15	3.06	0.00	0.24	0.87
	MED	30.9%	1.47	2.08	0.71	0.48	0.09	23.62
Total variance		100.0%	4.74					

Supplementary table 5
Parameter estimates by center for Expenditure incurrence
Adults

		% Variance	B	S.E.	Wald	Sig.	Exp(B)
Center A							
Fixed	age		0.19	0.02	88.25	0.00	1.21
	age squared		-0.04	0.01	29.06	0.00	0.96
	Intercept		1.87	0.03	3818.52	0.00	6.51
Variance		1.2%					
Random	MED	0.9%					
	ACG	32.9%					
	Residual	65.0%					
	Total	100.0%					
Center B							
Fixed	age		0.03	0.02	2.02	0.16	1.03
	age squared		-0.03	0.01	14.57	0.00	0.97
	Intercept		1.85	0.04	2513.28	0.00	6.33
Variance		0.3%					
Random	MED	1.2%					
	ACG	29.6%					
	Residual	69.0%					
	Total	100.0%					
Center C							
Fixed	age		0.11	0.02	34.47	0.00	1.12
	age squared		0.00	0.01	0.30	0.58	1.00
	Intercept		1.98	0.03	4859.30	0.00	7.24
Variance		0.5%					
Random	MED	1.3%					
	ACG	32.0%					
	Residual	66.2%					
	Total	100.0%					
Center D							
Fixed	age		0.24	0.03	82.97	0.00	1.27
	age squared		-0.05	0.01	23.58	0.00	0.95
	Intercept		1.88	0.04	2129.04	0.00	6.58
Variance		2.0%					
Random	MED	1.9%					
	ACG	33.7%					
	Residual	62.3%					
	Total	100.0%					
Center E							
Fixed	age		0.10	0.03	16.31	0.00	1.11
	age squared		0.01	0.01	1.46	0.23	1.01
	Intercept		1.89	0.04	2208.10	0.00	6.64
Variance		0.6%					
Random	MED	1.6%					
	ACG	21.3%					
	Residual	76.6%					
	Total	100.0%					

Supplementary table 6
Parameter estimates by center for Level of expenditure
Adults

	Parameter	% variance	Estimate	Std. Error	Wald	Sig.	95% CI lower	95% CI upper
Center A								
Fixed	Intercept		5.85	0.25	23.27	0.00	5.35	6.35
	age		0.58	0.02	33.84	0.00	0.55	0.61
	age squared		-0.01	0.01	-1.13	0.26	-0.02	0.01
Variance		6.8%	0.52					
Random	Residual	51.8%	3.97	0.05	73.55	0.00	3.86	4.07
	ACG	40.3%	3.09	0.61	5.03	0.00	2.09	4.56
	MED	1.1%	0.09	0.04	2.03	0.04	0.03	0.23
Total variance		100.0%	7.66					
Center B								
Fixed	Intercept		5.52	0.28	19.46	0.00	4.95	6.09
	age		0.71	0.02	36.82	0.00	0.67	0.74
	age squared		-0.04	0.01	-4.75	0.00	-0.06	-0.02
Variance		6.7%	0.57					
Random	Residual	52.6%	4.49	0.08	58.66	0.00	4.35	4.65
	ACG	39.3%	3.35	0.68	4.90	0.00	2.25	5.00
	MED	1.5%	0.13	0.08	1.67	0.09	0.04	0.41
Total variance		100.0%	8.54					
Center C								
Fixed	Intercept		6.02	0.23	26.02	0.00	5.56	6.49
	age		0.57	0.01	41.52	0.00	0.55	0.60
	age squared		-0.01	0.01	-2.15	0.03	-0.02	0.00
Variance		10.6%	0.82					
Random	Residual	50.2%	3.90	0.05	85.49	0.00	3.81	3.99
	ACG	38.3%	2.98	0.54	5.48	0.00	2.08	4.26
	MED	0.9%	0.07	0.03	2.24	0.03	0.03	0.17
Total variance		100.0%	7.78					
Center D								
Fixed	Intercept		6.04	0.24	24.92	0.00	5.55	6.52
	age		0.57	0.02	25.44	0.00	0.52	0.61
	age squared		0.00	0.01	-0.18	0.86	-0.02	0.02
Variance		6.1%	0.45					
Random	Residual	51.7%	3.81	0.07	55.25	0.00	3.68	3.95
	ACG	42.1%	3.10	0.63	4.94	0.00	2.08	4.61
	MED	0.1%	0.01	0.01	0.88	0.38	0.00	0.06
Total variance		100.0%	7.37					
Center E								
Fixed	Intercept		5.79	0.29	20.23	0.00	5.20	6.38
	age		0.65	0.02	33.21	0.00	0.61	0.69
	age squared		-0.03	0.01	-3.90	0.00	-0.05	-0.02
Variance		5.8%	0.46					
Random	Residual	56.5%	4.48	0.08	58.98	0.00	4.34	4.63
	ACG	35.4%	2.80	0.55	5.14	0.00	1.91	4.11
	MED	2.4%	0.19	0.12	1.55	0.12	0.05	0.66
Total variance		100.0%	7.93					

Supplementary table 7
Parameter estimates by center for Expenditure incurrence
Pediatrics

		% Variance	B	S.E.	Wald	Sig.	Exp(B)
Center A							
Fixed	age		-0.23	0.18	1.75	0.19	0.79
	age squared		-0.06	0.08	0.55	0.46	0.94
	Intercept		1.25	0.05	530.49	0.00	3.50
Variance		0.4%					
Random	MED	0.0%					
	ACG	43.8%					
	Residual	55.8%					
	Total	100.0%					
Center B							
Fixed	age		-0.44	0.14	9.78	0.00	0.64
	age squared		-0.01	0.22	0.00	0.95	0.99
	Intercept		0.38	0.06	44.32	0.00	1.46
Variance		0.9%					
Random	MED	19.2%					
	ACG	23.7%					
	Residual	56.2%					
	Total	100.0%					
Center C							
Fixed	age		-0.44	0.18	5.97	0.01	0.65
	age squared		0.10	0.43	0.06	0.81	1.11
	Intercept		1.85	0.06	811.80	0.00	6.36
Variance		0.4%					
Random	MED	0.3%					
	ACG	35.7%					
	Residual	63.7%					
	Total	100.0%					
Center D							
Fixed	age		0.30	0.26	1.39	0.24	1.36
	age squared		-0.86	0.60	2.07	0.15	0.42
	Intercept		-2.05	0.10	453.37	0.00	0.13
Variance		0.4%					
Random	MED	0.1%					
	ACG	16.6%					
	Residual	83.0%					
	Total	100.0%					
Center E							
Fixed	age		-0.54	0.16	10.99	0.00	0.58
	age squared		-0.26	0.37	0.48	0.49	0.77
	Intercept		-0.34	0.07	23.63	0.00	0.71
Variance		1.1%					
Random	MED	27.9%					
	ACG	17.1%					
	Residual	54.0%					
	Total	100.0%					

Supplementary table 8
Parameter estimates by center for Level expenditure
Pediatrics

	Parameter	% variance	Estimate	Std. Error	Wald	Sig.	95% CI lower	95% CI upper
Center A								
Fixed	Intercept		3.67	0.19	19.77	0.00	3.23	4.11
	age		-0.53	0.11	-4.74	0.00	-0.75	-0.31
	age squared		0.09	0.06	1.43	0.15	-0.03	0.22
Variance		0.8%	0.02					
Random	Residual	71.5%	1.54	0.06	27.38	0.00	1.43	1.65
	ACG	25.4%	0.55	0.17	3.28	0.00	0.30	0.99
	MED	2.3%	0.05	0.05	0.94	0.35	0.01	0.40
Total variance		100.0%	2.15					
Center B								
Fixed	Intercept		3.38	0.56	6.05	0.09	-2.46	9.21
	age		0.36	0.14	2.63	0.01	0.09	0.62
	age squared		-0.58	0.29	-1.97	0.05	-1.15	0.00
Variance		0.55%	0.02					
Random	Residual	68.3%	2.12	0.10	22.16	0.00	1.94	2.31
	ACG	12.1%	0.38	0.12	3.03	0.00	0.20	0.72
	MED	19.0%	0.59	0.84	0.70	0.48	0.04	9.67
Total variance		100.0%	3.10					
Center C								
Fixed	Intercept		4.36	0.21	21.14	0.00	3.93	4.79
	age		-0.16	0.09	-1.67	0.09	-0.34	0.03
	age squared		-0.52	0.27	-1.94	0.05	-1.05	0.00
Variance		0.6%	0.02					
Random	Residual	62.7%	1.98	0.05	36.36	0.00	1.88	2.09
	ACG	35.8%	1.13	0.30	3.82	0.00	0.68	1.89
	MED	0.9%	0.03	0.03	0.88	0.38	0.00	0.26
Total variance		100.0%	3.16					
Center D								
Fixed	Intercept		3.10	0.17	18.18	0.02	1.70	4.49
	age		0.43	0.34	1.26	0.21	-0.25	1.11
	age squared		-0.36	0.59	-0.60	0.55	-1.52	0.81
Variance		0.8%	0.01					
Random	Residual	96.0%	1.58	0.18	8.95	0.00	1.27	1.97
	ACG	1.3%	0.02	0.07	0.32	0.75	0.00	10.11
	MED	2.0%	0.03	0.07	0.45	0.65	0.00	2.52
Total variance		100.0%	1.65					
Center E								
Fixed	Intercept		3.49	0.14	25.69	0.00	3.20	3.77
	age		0.17	0.19	0.86	0.39	-0.21	0.55
	age squared		-0.33	0.47	-0.69	0.49	-1.25	0.60
Variance		0.4%	0.01					
Random	Residual	88.2%	2.30	0.14	15.94	0.00	2.04	2.60
	ACG	11.3%	0.29	0.18	1.63	0.10	0.09	0.98
	MED	0.0%	0.00	0.00	-	-	-	-
Total variance		100.0%	2.61					
